# The Context of Sexual Risk Behaviour Among Men Who Have Sex with Men Seeking PrEP, and the Impact of PrEP on Sexual Behaviour

**DOI:** 10.1007/s10461-018-2300-5

**Published:** 2018-10-10

**Authors:** Mitzy Gafos, Rob Horne, Will Nutland, Gill Bell, Caroline Rae, Sonali Wayal, Michael Rayment, Amanda Clarke, Gabriel Schembri, Richard Gilson, Alan McOwan, Ann Sullivan, Julie Fox, Vanessa Apea, Claire Dewsnap, David Dolling, Ellen White, Elizabeth Brodnicki, Gemma Wood, David Dunn, Sheena McCormack

**Affiliations:** 1 London School of Hygiene and Tropical Medicine, London, UK; 2 Medical Research Council Clinical Trials Unit, Institute of Clinical Trials and Methodology, University College London, London, UK; 3 School of Pharmacy, University College London, London, UK; 4 Prepster, London, UK; 5 Sheffield Health, Sheffield Teaching Hospitals NHS Foundation Trust, Sheffield, UK; 6 Chelsea and Westminster Hospital NHS Foundation Trust, London, UK; 7 Centre for Population Research in Sexual Health and HIV, Mortimer Market Centre, Institute for Global Health, UCL, London, UK; 8 Claude Nicol Centre, Royal Sussex County Hospital, Brighton, UK; 9 Manchester Centre for Sexual Health, Central Manchester University Hospitals NHS Foundation Trust, Manchester, UK; 10 The Mortimer Market Centre, Institute for Global Health, University College London, London, UK; 11 Guy’s and St Thomas’ NHS Foundation Trust, London, UK; 12 Ambrose King Centre, Barts Health NHS Trust, London, UK

**Keywords:** Pre-exposure prophylaxis, PrEP, HIV prevention, Risk compensation, Sexual risk behaviour, Gay and other MSM, UK

## Abstract

There are still important gaps in our understanding of how people will incorporate PrEP into their existing HIV prevention strategies. In this paper, we explore how PrEP use impacted existing sexual risk behaviours and risk reduction strategies using qualitative data from the PROUD study. From February 2014 to January 2016, we conducted 41 in-depth interviews with gay, bisexual and other men who have sex with men (GBMSM) enrolled in the PROUD PrEP study at sexual health clinics in England. The interviews were conducted in English and were audio-recorded. The recordings were transcribed, coded and analysed using framework analysis. In the interviews, we explored participants’ sexual behaviour before joining the study and among those using or who had used PrEP, changes to sexual behaviour after starting PrEP. Participants described the risk behaviour and management strategies before using PrEP, which included irregular condom use, sero-sorting, and strategic positioning. Participants described their sexual risk taking before initiating PrEP in the context of the sexualised use of drugs, geographical spaces linked with higher risk sexual norms, and digitised sexual networking, as well as problematic psychological factors that exacerbated risk taking. The findings highlight that in the main, individuals who were already having frequent condomless sex, added PrEP to the existing range of risk management strategies, influencing the boundaries of the ‘rules’ for some but not all. While approximately half the participants reduced other risk reduction strategies after starting PrEP, the other half did not alter their behaviours. PrEP provided an additional HIV prevention option to a cohort of GBMSM at high risk of HIV due to inconsistent use of other prevention options. In summary, PrEP provides a critical and necessary additional HIV prevention option that individuals can add to existing strategies in order to enhance protection, at least from HIV. As a daily pill, PrEP offers protection in the context of the sex cultures associated with sexualised drug use, digitised sexual applications and shifting social norms around sexual fulfilment and risk taking. PrEP can offer short or longer-term options for individuals as their sexual desires change over their life course offering protection from HIV during periods of heightened risk. PrEP should not be perceived or positioned in opposition to the existing HIV prevention toolkit, but rather as additive and as a tool that can and is having a substantial impact on HIV.

## Introduction

In 2012, the Food and Drug Administration (FDA) approved Truvada^®^ (tenofovir disoproxil fumarate and emtricitabine) as the first HIV pre-exposure prophylaxis (PrEP) drug, based primarily on evidence from three placebo controlled trials [1–4]. The concern about sexual risk compensation or disinhibition has accompanied the development of new HIV prevention technologies over the last 20 years, and was highlighted in the reflection paper issued by the European Medicines Agency [5, 6]. Initially, the concern was that a reduction in condom use in response to the uptake of partially effective options such as medical male circumcision, vaccines and microbicides, could lead to an increase in HIV exposure and consequently acquisition [5, 7]. As confidence in the biological efficacy of PrEP has increased, there is concern that a reduction in condom use could lead to an increase in STI acquisition and related STI drug resistance.

At the time of the FDA approval, there was no evidence of risk compensation in the reviewed trials [8, 9]. However, in placebo controlled trials participants did not know if they were using PrEP or if PrEP was effective and therefore would be unlikely to change their behaviours. As such, there were unanswered questions about the potential for risk compensation when people were using PrEP knowing it was efficacious against HIV acquisition. In order to support PrEP availability in England, the PrEP e-group, formed in 2011 of community members, healthcare providers, policy makers and researchers, recommended a randomised open label wait-listed trial design [10]. By randomising participants to an immediate start of PrEP or a deferred start of PrEP after 12 months, the trial design allowed for a robust assessment of differences in sexual behaviour and STIs between PrEP and non-PrEP users.

The PROUD (**PR**e-exposure **O**ption for reducing HIV in the **U**K: immediate or **D**eferred) trial demonstrated that the inclusion of PrEP in the sexual health package offered to gay, bisexual and other men who have sex with men (GBMSM) and trans women, reduced HIV acquisition by 86%, with no infections among participants taking PrEP at the likely time of exposure [11]. The offer of PrEP in the PROUD trial attracted a high-risk group of GBMSM evidenced at baseline by high numbers of condomless sex partners (with condomless sex being an eligibility criteria) and two-thirds of participants reporting an STI in the previous 12 months, and during follow up by an HIV incidence of 9 per 100 persons years in the control group [11, 12]. The PROUD trial did not attract trans-women, with only three enrolling, probably due to limitations of the recruitment strategy.

In the main trial analysis, although there were no differences between the groups in terms of most sexual behaviour, a larger proportion of participants allocated to the immediate PrEP than to the deferred PrEP group reported receptive condomless anal sex with ten or more partners. Despite this, there was no statistically significant difference between the groups for STIs overall, after adjusting for the difference in the number of STI screens during the first year of follow up [11]. The conclusion from the trial was that among high risk GBMSM reporting regular condomless sex, the offer of PrEP dramatically reduced HIV acquisition, and did not impact on STIs despite a suggestion of lower condom use among a small proportion of PrEP users.

PrEP is now available in over 20 countries globally and implementation programmes are increasing across Europe. While PrEP is available as standard of care in Scotland and Wales, it is currently only available for free through participation in the Public Health England PrEP Impact trial in England, or via private purchase. While the benefit of PrEP as a HIV prevention method is clear, there are still gaps in our understanding of how people incorporate PrEP into their existing HIV prevention strategies. In this paper, we explore how PrEP use impacted existing sexual risk behaviours and risk reduction strategies using qualitative data from the PROUD study.

## Methods

PROUD was a pragmatic open-label wait-listed randomised controlled trial designed to evaluate the effectiveness of daily oral Truvada as HIV PrEP. Recruitment took place from November 2012 to April 2014 at 13 sexual health clinics in England, eight in London and five outside of London. Eligibility criteria included HIV negative GBMSM or trans-women, aged 18 or above, who reported condomless anal sex in the last 90 days and expected to have it again in the next 90 days. At enrolment, eligible participants were randomised 1:1 to receive PrEP immediately or after a deferred period of 12 months. Participants were asked to attend the clinic quarterly for at least 2-years. The study design, baseline characteristics of the cohort and results have been reported elsewhere [11, 12].

From February 2014 to January 2016, we planned to purposefully sample up to 50 study participants to take part in semi-structured in-depth interviews (IDIs). We aimed to select 44 participants based on trial arm allocation (immediate or deferred), changes in their self-reported risk behaviour since enrolment (increased risk or same/decreased risk), and self-reported PrEP adherence among participants in the immediate group (high or low). From September 2015, we amended the selection criteria slightly in an attempt to identify more variability by risk behaviour, thereby selecting participants based on self-reported current risk behaviour instead of changes since enrolment (high risk or low/medium risk). In addition to the six selection categories we aimed to purposefully select approximately six individuals to explore under-represented topics.

Researchers who were independent of the study clinic team conducted the IDIs. The IDIs lasted on average between 45 and 60 mins and participants were not compensated for their time. The IDI guide included a discussion topic on sexual risk behaviour, as well as perceptions, experiences and usage of PrEP. Participants provided written informed consent and the IDIs were conducted in English and were audio-recorded. The recordings were transcribed, coded and analysed using framework analysis in NVivo 10 [13]. The first author coded the interviews, each interviewer reviewed the coding of a sub-sample of their interviews, and coding discrepancies were discussed. In the results section, we present quotes that illustrate the key findings and identify participants by selection criteria (trial arm, risk behaviour, if on PrEP by adherence), whether they had used PrEP by the time of the interview, whether they were enrolled in a clinic in or out of London, and the age group there were in at enrolment.

The PROUD study protocol was approved by London Bridge Research Ethics Committee, the Medicines and Healthcare Products Regulatory Agency and each of the 12 participating Hospital Trusts (see list in acknowledgments). The trial is registered with ISRCTN (Number ISRCTN94465371) and ClinicalTrials.gov (NCT02065986). The study protocol, including participant information sheet (PIS) and informed consent form (ICF), and the in-depth interview PIS, ICF and interview guide, are available on the study website (www.proud.mrc.ac.uk).

## Results

**Fig. 1:**
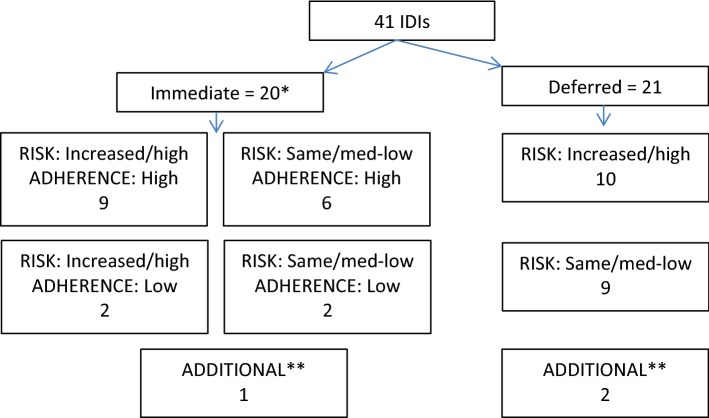
Purposeful selection of participants for in-depth interviews based on trial arm allocation, risk behaviour and adherence. *One participant co-enrolled and in the main trial analysis is treated as being in the deferred group as this was his original allocation. However, he is treated as being in the immediate group here as that is how he was selected for IDI. **Additional interviews were conducted with a trans-woman, a person who sero-converted during the study, and a person who decided not to start PrEP

We interviewed a total of 41 study participants. Thirty-eight were selected equally from the immediate and deferred control groups. We conducted three additional interviews to explore under-represented topics, one with a trans woman, another with a person who sero-converted during the study, and another with a person who decided not to start PrEP (Fig. 1). By the time of interview, 33 participants told us they were or had been using PrEP. Thirty participants had been prescribed PrEP in the trial, although two had stopped using it by the time of the interview. A further three had accessed PrEP privately, one by using the Truvada from PEP and another using Truvada from a HIV-positive partner both during the deferred period, and a third who had purchased it in the USA and used it before joining the trial. Participants had used PrEP for a mean of 14.3 months by the time of the interview, ranging from one week to 32.8 months. Due to high adherence across the study cohort, we were unable to interview the planned numbers of participants who reported low adherence. We interviewed participants from five clinics in London, as well as clinics in Sheffield, Manchester and Brighton. We present the baseline demographic and sexual behaviour profile of the participants who were interviewed in Tables 1 and 2.

**Table 1:** Demographic and behaviour at enrolment

Median age (interquartile range)	37.4 (31.9, 42.7)
*Clinic of enrolment*	
London	24
Sheffield	9
Manchester	5
Brighton	3
*Ethnicity*	
White/Irish	34
BAME^a^	7
*Place of birth*	
UK	26
Other^b^	15
*University degree education*	
Yes	25
No	16
*Employed*	
Yes	36
No	5
*In a relationship*	
Yes	17
No	24
*Sexuality*	
Gay	40
Bi-sexual	1
*Gender*	
Cis-male	40
Trans-female	1
*Circumcised*	
Yes	10
No	31
*Symptoms of depression*	
Yes	6
No	35
*Chemsex use in the past 3 months*	
Yes^c^	13
No	28
*PEP use in last year* ^d^	
Yes	14
No	26
*Self-reported STI in last year* ^e^	
Yes	18
No	21

^a^BAME ethnicities include Pakistani, Hispanic, Arabic, mixed ethnicity

^b^Other includes Australia, South America, South Africa and the rest of Europe (1 missing)

^c^Chemsex use includes 12 participants using GHB, 9 using methedrone, and 7 using crystal meth

^d^PEP use excludes one missing response; six participants used PEP more than once

^e^STIs in last year excludes two missing response

**Table 2:** Sexual behaviour in last 90 days, reported at enrolment

Median number of anal sex partners (interquartile range)	10 (3, 20)
*Had anal sex with a new partner*	
Yes	35
No	6
*Been passive partner*	
Yes	39
No	2
*Been passive partner and condomless*	
Yes	37
No	4
*Been passive during condomless sex with HIV positive man*	
Yes	14
No	27
*Been passive during condomless sex with HIV positive man who you didn’t know was on treatment*	
Yes	1
No	40
*Been active partner*	
Yes	40
No	1
*Been active partner and condomless*	
Yes	40
No	1
*Been active during condomless sex with HIV positive man*	
Yes	20
No	21
*Been active during condomless sex with HIV positive man who you didn’t know was on treatment*	
Yes	2
No	39

In the interviews, we explored participants’ sexual behaviour before joining the study and among those using or who had used PrEP, changes to sexual behaviour after starting PrEP. Below we present data on three key themes: risk behaviour and management strategies before using PrEP, the contexts within which risk taking occurs, and the impact of PrEP on sexual behaviours.

### Risk Behaviour and Management Strategies Before Using PrEP

All participants recognised they were taking risks that potentially exposed them to HIV. A few participants underestimated the risk of the behaviour they reported and many rationalised their risk taking:

“Here I am quantifying different risks at the high risk end of the spectrum, and I know there are different levels of risk in the high risk end, although I am not making the most sensible decisions I think I am at the lower end of the higher risk end, er.. sometimes anyway” (Deferred, decreased risk, not on PrEP, London clinic, age 35-39).

Participants often described their risk reduction strategies in terms of the ‘rules’ that they applied to their sexual activity before they started using PrEP, which included condom use, strategic positioning, sero-sorting, avoiding ejaculate, and the use of PEP, as described below.

#### Condoms

Most participants reported using condoms inconsistently with rules about when and with whom to use them. Only four participants reported never using condoms and two reported consistent condom use with their main partner. The main reasons cited for not using condoms were that they reduced sexual pleasure and intimacy. A number of participants described how and why they had decided to not use condoms:

“The use of a condom is really inhibiting my sexual pleasure, being passive but especially being active, and one and a half years ago I decided to stop being afraid, and that doesn’t mean I am a bug chaser… I would like to postpone (acquiring HIV) as long as possible, but what I absolutely want is not to be afraid anymore… you may think well why are you doing this, well because it is impacting how I experience sex and how I experience my sexuality” (Deferred, decreased risk, not on PrEP, London clinic, age 35-39).

#### Strategic Positioning

Participants were aware of strategic positioning as a risk reduction strategy in terms of receptive anal sex carrying more risk of infection than insertive anal sex. Most participants reported being versatile in their sexual positioning, thereby reporting being both receptive (passive) and insertive (active) during sex largely depending on the type of partner they were having sex with. Many applied their own ‘rules’ to when they would be the active or passive partner during sex, for example only being receptive in a relationship or not being receptive during condomless sex. However, there were frequent examples of participants not applying their own ‘rules’ particularly when under the influence of alcohol or drugs:

“I wouldn’t be passive for anyone, and this was obviously when I was compos mentis, because obviously when I was pissed or on a rare occasion, I would you know just be passive” (Deferred, low risk, on PrEP, out of London clinic, age 25-29).

#### Sero-Sorting

Most participants discussed HIV status with potential partners before sex or viewed the HIV status of potential partners online if meeting via sexual networking apps. The majority were aware that viral suppression reduced the risk of HIV transmission. Only one participant exclusively selected negative partners on dating sites. A handful of participants reported exclusively having condomless sex with HIV positive men who had undetectable viral loads. However, the limitation of sero-sorting as a HIV prevention strategy was acknowledged whereby a positive person may be undiagnosed, may not be truthful about their status, or may not be sufficiently adherent to treatment to remain undetectable:

“I tend to only play bareback with undetectable guys who I know are kind of trustworthy and taking their medications, they aren’t absolute drug heads who go on benders for weekends and don’t take their meds” (Deferred, increased risk, on PrEP, London clinic, age 25-29).

#### Ejaculation

About half a dozen participants mentioned rules around ejaculation as part of their risk reduction strategies, for example not ejaculating or receiving ejaculate during condomless sex, or only doing so within a relationship.

#### Post Exposure Prophylaxis (PEP)

Almost half of the participants interviewed reported using PEP before the study. A couple of participants described accessing PEP after being raped and a couple after unplanned potential exposure with positive partners. However, besides these clear cases it appeared that participants’ decisions to access PEP were not consistent given equivalent risks. Some participants acknowledged that there were multiple occasions when they should have probably accessed PEP but did not. A few participants described how they didn’t access PEP due to a sense of guilt and shame:

“Some of the PEPs were coming close to each other and there were times that I should have been coming in but was too embarrassed because I had it quite recently” (Immediate, high adherence, decreased risk, on PrEP, London clinic, age 40-49).

Only one participant described feeling ‘judged’ by health care workers when trying to access PEP:

“One member of staff said to me at one point ‘Well you say all these things, these wonderful things, but then you end up back and doing it again’ … that’s when you think that you can’t go back” (Deferred, high risk, on PrEP, out of London clinic, age 35-39).

Many participants who hadn’t used PEP, described circumstances where they probably should have accessed it and would have met the eligibility criteria for it. Participants clearly only perceived PEP as an emergency response to a specific incident and not as a regular HIV prevention option.

#### Limited Risk Reduction

Not surprisingly, not everyone applied ‘rules’ to their sexual activity and many struggled to apply the rules they had set for their own behaviour. For some, the decision on whether or not to use a condom with a new partner sometimes relied on instinct:

“It depends on how you have met them and how they come across and you just go with your gut on that” (Deferred, increased risk, on PrEP, London clinic, age 35-39).

For others, the decision of whether or not to use a condom largely relied on trust, even with casual sexual partners. This seemed to mainly emerge in the interviews outside of London and Brighton, whereby participants tended to trust that the ‘friends’ who they had regular casual sex with, would not put them at risk and therefore expected them to use condoms with ‘other’ partners. As such they considered their friends a safe group with whom to have condomless sex and in a few cases, were genuinely surprised when they contracted an STI from them.

### Contexts of Risk Taking

Most participants described their risk taking within social, cultural and environmental contexts of the ‘gay scene’. When describing the contexts within which they took risks, we identified three key themes, that of risks associated with the sexualised use of recreational drugs (defined as the use of illicit drugs just before or during sexual activity), risks associated with certain geographical spaces, and the use of sexual networking apps as a means to access sex and risk taking. In addition, participants described psychological factors associated with sexual risk. We present each of these contexts below.

#### Sexualised Use of Recreational Drugs

The sexualised use of recreational drugs was a common theme when discussing the various contexts within which risk taking occurred. This was commonly referred to as ‘chemsex’ which is a colloquial term for the use of GHB/GBL (commonly called G), crystal methamphetamine (commonly called T or tina), or Mephedrone (commonly called meow meow or M-cat) before and during sex. Some participants also talked about using Ketamine during sex.

Almost half of the participants interviewed reported engaging in chemsex and described the experience as increasing sexual desire and a fixation on sexual fulfilment, increasing sexual disinhibition, and intensifying the sexual experience. All participants who engaged in chemsex described increased risk taking in this context:

“The drug that has made the biggest difference to my sexual behaviour is mephedrone… It’s a cheap drug which has become more readily available around London over the last couple of years and I have noticed that for me it has a high association with bad methods of protection whereby I will do much more reckless things … I would identify that for me, as a particular drug associated with chemsex and high-risk behaviour” (Immediate, high adherence, low risk, on PrEP, London clinic, age 45-49).

#### Geographic Spaces

Cities like London, Brighton and Manchester, where most of the participants lived, were frequently described as being ‘risky’ on the basis of higher HIV prevalence, accessibility of gay venues, a shift in social norms related to condoms, and as the epicentres of the growing urban chemsex gay scene. It is not practical to compare participant experiences between the London, Brighton, Manchester and Sheffield clinics due to the small numbers interviewed outside of London. However, there was a sense in the Sheffield interviews that participants’ risk taking was lower than described in the interviews conducted in other cities. This seemed to partly relate to different social norms relating to the acceptable level of risk taking and to the prominence of chemsex on the gay scene.

“I have moved to Brighton from a different part of the country. I have had a bit of a culture shock. There have been times that I have actually felt that I almost needed to get away from Brighton because it seems to be a bit of a hot bed of unprotected sex, substance abuse and I know that it is a bit of a HIV hotspot” (Deferred, increased risk, on PrEP, out of London clinic, age 35-39).

#### Sexual Networking Apps

Similarly, the socio-cultural environment of sexual networking applications such as Grindr, Gaydar, Scruff, BBRT and RECON, was described as fuelling both sexual contacts, identification of partners for condomless sex, and access to drugs and sex parties:

“If you log onto Grindr, you look through these things and there are quite a few instances which I feel like I see more often more recently …. Of people talking about meth and group sessions and G and all this kind of stuff “(Deferred, increased risk, on PrEP, London clinic, age 20-24).

#### Psychological Factors

In the interviews, about a quarter of participants specifically linked problematic psychological factors to their risk taking. These factors included post-traumatic stress, bereavement, psychological breakdowns, relationship breakups, and a history of depression and anxiety. In a number of cases, psychological problems fuelled, and were fuelled by, both chemsex and sexual networking apps:

“For 20 plus years I had perfect adherence to condoms. Then about 4 or 5 years ago there were two things that happened, well three really. Firstly, I noticed that sexual behaviours were changing around me with far less condom use… real generational issue… Secondly, I went through a number of bereavements and started experiencing depression. This impacted on my sexual behaviour and I started having more unsafe sex. Thirdly, I started using drugs. So, it was all those three things that resulted in me having unsafe sex and seeking out the PROUD study” (Immediate, high adherence, low risk, on PrEP, London clinic, age 45-49).

### Impact of PrEP on Sexual Behaviour

By the time of the in-depth interviews, 33 participants were or had been using PrEP. We asked participants directly whether they had changed their sexual behaviour since using PrEP, as well as comparing the sexual behaviour that participants described before and after starting PrEP.

Participants described the ways in which starting PrEP had impacted on their sexual behaviour along a continuum from changes being part of general fluctuations in behaviour, to reporting noticeable changes in their behaviour since starting PrEP, to not changing their existing behaviours. We present data on these three categorisations below.

#### Fluctuating Patterns of Sexual Behaviour

Most participants described fluctuating patterns of sexual risk taking over the course of their lives. The patterns were influenced by age, stage of life (for example when in university), movement to a new job, home or city, changes in relationship status with the end of relationships often triggering peaks in risk taking, social movement in and out of higher risk sex scenes such as the chemsex scene, as well as changes due to psychological factors.

In this context of fluctuating patterns of risk, a number of participants had recognised changes in their behaviour but were unsure if the changes merely coincided with them starting PrEP:

“Since I started PrEP, this is a complete coincidence, my sexual life had died off a bit, I’d been through a bit of a depression for the last couple of years, nothing to do with the PrEP it’s just my life had taken a few turns for the worse basically and so sexual relations had died off for me” (Immediate, low adherence, high risk, on PrEP, out of London clinic, age 30-34).

“This is the really bizarre thing, whereas I was being passive very often with a number of people who were acquaintances … but since PrEP I have actually only been passive with one person in the last year, on multiple occasions but even so I thought with PrEP oh yes I’ll be able to have sex as much as I want now but it’s not kind of logical like that, I just started taking it at a time when I was taking a lot of risks, and my life for whatever reason has gone down a less risky path… I’ve bottomed less, and with less partners” (Deferred, low risk, on PrEP, out of London clinic, age 25-29).

Other examples of initial unexpected decreases in sexual risk behaviour appeared to be more directly in response to starting PrEP:

“At first, I think I stopped having sex, I can’t quite explain that. When I first started taking PrEP it was a couple of months before I really had anything penetrative… it might be coincidence… I think part of it was being a bit over aware of my behaviour and whether it was going to change… and it could just be that I was getting paranoid… as time passed I was clearly less worried about condom use… they were never great for me, but I used them intermittently” (Immediate, high adherence, high risk, on PrEP, London clinic, age 40-44).

Conversely a few other participants described spikes in risk taking when they started PrEP, which dissipated over time as their personal circumstances and relationships changed, sometimes linked to drug use as in this quote:

“At some subconscious level, I think it’s supporting me to get to that relationship, and the unprotected sex went down after it spiked… it all spiked and then suddenly it tailed off, I had this relationship and it’s not really spiked back up since. I’ve not used drugs for about five months now” (Deferred, high risk, on PrEP, out of London clinic, age 20-24).

There were other examples of the peaks and troughs of sexual activity, such as this participant whose risk taking was very low when he started PrEP due to entering a new relationship, very high when the relationship ended, and by the time of the interview had returned to what it was prior to starting PrEP:

“There used to be a point where… somebody was like ‘oh we are going to have safe sex’ and I was like ‘no I don’t want it’… whereas now I’d be thinking yeah okay that’s totally fine, because I want to meet somebody where you start, and you have the discussions and you have safe sex and then through time … if you want to have unprotected sex … that they know …I’m negative and… that this is somebody who is maybe responsible and they want a long term relationship” (Deferred, increased risk, on PrEP, London clinic, age 25-29).

While most participants perceived PrEP as a temporary risk reduction strategy for periods in their life of heightened risk, a couple of participants described it in the context of an immediate release from an overwhelming need for sexual freedom:

“I was in an awful place and I just felt like I was holding the lid on a boiling pot of water so hard that I just couldn’t hold it anymore (trying to avoid sex without condoms)… so I started on PrEP in the middle of March … and in that time … I feel like I have come full circle, I feel like I have slowly released that pressure on that pot of water to the point that it’s OK now, it is just simmering it’s fine, I have had all my release that I needed to and I have almost come full circle that I don’t feel that strange obsessional necessity for bareback sex like I was before” (Deferred, increased risk, on PrEP, London clinic, age 25-29).

#### Changes in Sexual Behaviour Since Using PrEP

Overall, approximately half of all participants who had used PrEP described ways in which they had changed their sexual behaviour since using PrEP. Some participants described how they had previously struggled to use condoms, and therefore the desire to reduce condom use precipitated their use of PrEP:

“It is a question of pushing against an open door, clearly I wanted to participate in unsafe sex and obviously because of the risk I have had some resistance to unsafe sex, obviously not entirely 100%. But in taking the medication the levels of anxiety over that diminished. So where I may have hesitated previously, I dont hesitate any further…. But even now there is a bounded risk analysis” (Immediate, high adherence, increased risk, on PrEP, London clinic, age 50-54).

In the majority of cases participants added PrEP to their existing set of rules and PrEP influenced these rules in complex and often subtle ways. For some participants, using PrEP meant that they reduced their use of condoms or changed their rules about when to use condoms:

“There is no doubt that I have also had more unprotected sex than before, for sure…it doesn’t mean that I only have unprotected sex, but it does mean that I have more… it is probably 80% more, much more than protected” (Deferred, high risk, on PrEP, out of London clinic, age 45-49).

“I felt like it gave me the green card to do whatever the hell I wanted…I don’t use condoms at all any more. I’m starting to notice that its actually bad where, not that I’m encouraging people to not use condoms with me, but it’s like if I’m going to be a top there is no point in me wearing a condom, because I just can’t do it anymore, it won’t work so if they want to have sex with me, then it’s a level of risk that they have to accept and I won’t push it on them, but I can’t do it anymore (use condoms). *And I don’t know whether I like that development*” (Immediate, high adherence, high risk, on PrEP, London clinic, age 30-34).

As with the last sentence in the quote above, a few participants were not comfortable with their reduced condom use. However, this discomfort was not about the risk of STIs but seemed to be solely related to social norms about condom use for gay men. The following quote is another example of this discomfort when asked by the interviewer “Have any of your decisions around sex changed?”

“Yes I would say so and I am not always convinced it has been for the better… So there have been a couple of occasions where I haven’t used condoms with completely random one-off partners … when I have felt pretty shitty for some reason or another, and I have acted out sexually” (Immediate, low adherence, low risk, on PrEP, London clinic, age 30-34).

Other participants reported the main benefit of using PrEP was the opportunity to stop using condoms with HIV positive partners within relationships, despite understanding the evidence related to an undetectable viral load:

“I have a partner that is HIV positive, we have gone without condoms since being on the study, which we wouldn’t do before” (Immediate, high adherence, increased risk, on PrEP, London clinic, age 35-39).

“We don’t use condoms since I’ve been on PrEP… he’s undetectable anyway and with me being on PrEP, so no we don’t use condoms anymore… that is something we still don’t do, we don’t cum in one another, it would just seem silly given the circumstances” (Immediate, high adherence, low risk, on PrEP, out of London clinic, age 40-44).

For some participants PrEP use influenced the ‘rules’ that they had previously applied to their sexual activity in terms of sero-sorting and strategic positioning:

*“*It has definitely made me more likely to take risks, definitely I must admit, as I don’t tend to worry about what the status of the person is … now” (before PrEP, sero-sorted for negative partners and never used condoms) (Deferred, decreased risk, on PrEP, out of London clinic, age 35-39).

“I have always been more top, however it is true that I have definitely experienced more as a bottom since (PrEP)… now I am more relaxed about letting a guy fuck me, I just don’t desire it that much, it takes a special kind of person to make me want to do it” (Deferred, high risk, on PrEP, out of London clinic, age 40-44).

Although some participants did not think that PrEP had influenced their behaviour, they described sex they were not sure they would have had without PrEP:

“I have a friend … we are effectively dating but there has been no attempt at a long-distance relationship. We use condoms but at the end of this trip … I was like, okay I know you have been tested recently, I know you have been on PrEP as well… and I thought I see you so rarely it would be fun to have unprotected sex… I knew it was a risk and at the end of the three weeks I thought I’m going to have unprotected sex with this guy and I did … I can’t remember any other occurrence where I made an active decision” (Deferred, low risk, on PrEP, out of London clinic, age 25-29).

#### Consistent Sexual Behaviour Before and After PrEP

While approximately half of participants reported changing their sexual behaviour since starting PrEP, the other half firmly believed that using PrEP had not altered their sexual behaviour.

“I haven’t changed the way I think because I am taking this pill… having these pills doesn’t give me an excuse to be more crazy than I already am” (Immediate, high adherence, increased risk, on PrEP, London clinic, age 35-39).

For most of the participants who didn’t change their behaviour, PrEP was the ‘additional’ protection that they added to their existing risk reduction strategies in an attempt to minimise the risk of HIV acquisition while having the sex that they desired:

“I’ve taken the medication and obviously I’ve still had intercourse in the way that I would if I’ve not taken the tablet really, so it’s not done anything with the behaviour side of it, my behaviours have remained the same throughout the full 24 months of me taking the tablet, I’d still behave in the way that I would” (estimates he has condomless sex with about 10% of partners) (Immediate, high adherence, low risk, on PrEP, out of London clinic, age 20-24).

In some cases, participants reported that their risk taking was already substantial and therefore did not increase after starting PrEP:

“I was having a huge amount of condomless sex before PrEP… I am not going to not bareback and always use condoms” (Immediate, high adherence, decreased risk, on PrEP, London clinic, age 45-49).

For others, their risk taking was relatively low before they started using PrEP and it remained so:

“I have a partner who is positive… and undetectable… prior to going onto this programme anyway we had been having unprotected sex, so for me it is more a belt and braces thing” (Deferred, low risk, stopped PrEP, London clinic, age 50-54).

It is also worth pointing out that many of the interviews occurred before the release of the PROUD results. As such, while most participants were using PrEP on the basis of knowing that it reduced the risk of HIV, there is a chance that a few participants continued to circumscribe their behaviour within the context of a ‘trial’.

“I don’t know if it works, I’m hoping it does, but I’m not going to change and be more reckless because of it” (Immediate, low adherence, high risk, on PrEP, out of London clinic, age 30-34).

“If you said Truvada was 100% effective, I would abandon condom usage I think because as I said all of the other STIs are treatable” (condom use reduced after starting PrEP) (Deferred, high risk, on PrEP, out of London clinic, age 45-49).

In relation to this last quote, it was clear throughout the interviews that participants were concerned about HIV but largely viewed STIs as a treatable problem. A few participants were particularly concerned about Hep C mainly in relation to fisting and sex toys, but in the main the aim was to avoid the risk of HIV acquisition with little concern for other STIs. Considering this, it was noteworthy that in the majority of the interviews participants still referred to sex on PrEP without a condom as ‘unprotected’ sex and therefore did not change their language in terms of defining this as condomless sex protected by PrEP, at least in terms of HIV.

## Discussion

This is the first paper to qualitatively explore the impact of PrEP use on the sexual behaviour of gay, bisexual and other MSM in the UK. It contributes to a growing literature of the experiences of ‘first wave’ PrEP users [14]. By exploring the background context of sexual risk taking, we are able to describe the diverse ways in which the initiation of PrEP use influences sexual behaviour and risk management strategies. The findings highlight that in the main, individuals who were already having frequent condomless sex, added PrEP to the existing range of risk management strategies, influencing the boundaries of the ‘rules’ for some but not all. While approximately half the participants reduced other risk reduction strategies after starting PrEP, the other half did not alter their behaviours. PrEP provided an additional HIV prevention option to a cohort of GBMSM at high risk of HIV due to inconsistent use of other prevention options.

This analysis highlights the complexities and changing dynamics of sexual risk behaviours for GBMSM in urban centres in England. PROUD participants self-identified as ‘at risk’ individuals and were all having condomless sex to varying degrees. Most participants attempted to manage their risk of HIV through an imperfect combination of risk reduction strategies including decisions around condom use, strategic positioning, sero-sorting, ejaculation and PEP use. This evidence is consistent with the quantitative findings from PROUD as well as qualitative findings from other PrEP studies in the USA [12, 15, 16]. All participants interviewed acknowledged that their existing risk management strategies were insufficient and therefore sought out PrEP as an additional risk management option.

Participants described their sexual risk taking before initiating PrEP in the context of the sexualised use of drugs, geographical spaces linked with higher risk sexual norms, and digitised sexual networking. In a recent qualitative study in England, newly diagnosed MSM identified these same three factors, drug use, geographical space and digital networking, as part of a complex web of factors that influenced their risk behaviours and HIV acquisition [17]. There is a growing body of evidence on the sexualised use of drugs in the UK and associations with higher risk sexual behaviours including condomless sex, multiple partners and group sex [18–23]. Indeed, in a European wide survey of MSM, London, Brighton and Manchester were the three cities with the highest rates of recent chemsex use (13.2–16.3%) [24]. In PROUD, 44% (231/525) of participants reported using any of the three drugs most commonly associated with chemsex in the three months prior to enrolment [12]. In the IPERGAY study in France, 29% (95/331) of participants reported chemsex and interestingly chemsex users were twice as likely to use PrEP the last time they had sex than non-users [25]. Evidence from both trials, and from a recent evaluation of PrEP use among chemsex users in Australia, suggests that MSM engaging in chemsex are incorporating PrEP into their sexual practice [26, 27]. PrEP, particularly event-based dosing, could play an important role in reducing the risk of HIV during periods in peoples’ lives when they are engaging in chemsex and related higher risk behaviours.

About a quarter of participants described their risk taking in the context of psychological factors. Problematic psychological factors are known to influence sexual risk-taking behaviour and individual’s ability to mitigate risk [28]. Psychological traumas are known to be an element of the multiple interrelated structural, social and biological factors that underpin risk taking [29]. Childhood and adult experiences of life stressors, trauma or discrimination have been shown to influence risk taking and HIV acquisition [17]. Indeed, in iPrEX, they observed an association between depression and higher risk sexual behaviour and specifically recommended the continued use of PrEP during periods of depression [30]. PrEP appears to offer an important layer of protection from higher risk sex during periods of depression or psychological stress.

In this study, the impact of PrEP on sexual behaviour was diverse, having a substantial impact on sexual behaviour for some participants and no or limited impact for others. The reality for most participants was that the inconsistency of their condom use in higher risk sexual contexts meant that PrEP was an important addition irrespective of the shifts in other prevention strategies. These qualitative data provide a more in-depth view of the nuances of sexual risk behaviour and the dynamic and fluid nature of risk, that was impacted by individual, inter-relational, social, cultural and psychological factors for many. Despite the continued focus on the potential public health harm of ‘risk compensation’ among this cohort of GBMSM, PrEP offered substantial protection against HIV as the biggest risk that they face in terms of sexual wellbeing and few participants prioritised protection from STIs to the same extent.

Evidence on the impact of PrEP on sexual behaviour is beginning to emerge although without a control group in a randomised control trial, it is difficult to measure impact during implementation programmes. Much of the quantitative research suggests few changes in sexual behaviour among PrEP users. For example, a systematic review and meta-analysis of data from 18 PrEP studies consistently found no reported decrease in condom use among participants using PrEP and with the exception of one trial, no reported increase in the number of sexual partners [31]. Similarly, in a wait-listed trial design in the USA, there were no differences in sexual behaviour between the PrEP and no-PrEP group, nor after initiation of PrEP in the deferred group [32]. A review of counselling notes in a PrEP clinic in San Francisco found that clients rarely reported changing existing sexual behaviours and in the main included PrEP in their existing HIV prevention strategies, which as in this paper, included intermittent condom use and sero-sorting [33]. In a qualitative study in the USA, while some participants acknowledged engaging in sexual encounters that they may have avoided prior to using PrEP, most incorporated PrEP into existing practice [15]. Similarly, in the iPrEX open label qualitative study, most participants did not substantially change their behaviour and the changes that did occur were usually fluid [16]. However, data from the open-label phase of IPERGAY and two PrEP implementation sites in San Francisco suggest decreases in condom use, although there is no evidence of an increase in STIs [34–37].

The strength of this analysis is that we collected qualitative data from individuals who were using PrEP in a randomised open-label study to reduce their risk of HIV acquisition. The fact that the qualitative data collection was integrated within the trial protocol, selection of interviewees was purposeful and derived from the self-reported quantitative data are also strengths of this study. The limitations are that the individuals in PROUD were a particularly high-risk group and clearly acknowledged their risk behaviours and as such may not be representative of all GBMSM in England who are seeking PrEP. Similarly, participants in PROUD were already inconsistent condom users, and therefore these findings do not address the impact of PrEP on condom use among GBMSM who consistently use condoms. In addition, the PROUD cohort were in the main highly educated, employed and predominantly white GBMSM, and therefore further research is required in order to speak to the needs of less privileged and marginalised groups of GBMSM as well as trans women [12]. Also of note, trans men were excluded from this study and are likely to have very different experiences of risk management. While the purposeful sampling approach had strengths in terms of allowing us to identify individuals based on their risk and adherence behaviour, it also had limitations as it meant we only selected participants who were completing the self-reported sexual behaviour questionnaire and therefore were most engaged in the study. As with all qualitative data, these data are subject to social desirability bias, although the descriptions of sexual risk taking in the interviews would suggest limited censoring of reporting risk behaviours. In this paper, we have focused exclusively on the impact of PrEP on sexual behaviour and this has enabled us to explore this topic in-depth. However, due to paper length we have been unable to describe participants’ experiences of using PrEP and the psychosocial benefits it offered. These data will be presented in separate papers.

In summary, PrEP provides a critical and necessary additional HIV prevention option that individuals can add to existing strategies in order to enhance protection, at least from HIV. As a daily pill, PrEP offers protection in the context of sex cultures associated with the sexualised use of drugs, digitised sexual applications and shifting social norms around sexual fulfilment and risk taking. PrEP can offer short or longer-term options for individuals as their sexual desires change over their life course offering protection from HIV during periods of heightened risk. PrEP should not be perceived or positioned in opposition to the existing HIV prevention toolkit, but rather as additive and as a tool that can and is having a substantial impact on HIV.
